# The “five-second” rule for dropped food: does it apply to dropped medical objects in the operating room? A randomized study of disinfection approaches for contaminated arthroplasty implants

**DOI:** 10.1017/ice.2026.10418

**Published:** 2026-05

**Authors:** Bobby Glenn Warren, Samuel Rosas, Eduardo Villoch, Amanda M. Graves, Aaron Barrett, Guerbine Fils-Aime, Christina Thomas, Thorsten Seyler, William Jiranek, Sean Ryan, Deverick J. Anderson, David Weber, Jessica Seidelman

**Affiliations:** 1 Disinfection, Resistance, and Transmission Epidemiology (DiRTE) lab, Durham, NC, USA; 2 Duke Center for Antimicrobial Stewardship and Infection Prevention, Durham, NC, USA; 3 Division of Infectious Diseases, Duke University Medical Center, Durham, NC, USA; 4 Department of Orthopaedic Surgery, Duke University Medical Center, Durham, NC, USA; 5 Duke University Health System, Durham, NC, USA; 6 Division of Infectious Diseases, University of North Carolina Health System, Chapel Hill, NC, USA

## Abstract

Polyethylene liners dropped onto operating room floors rapidly acquire bacterial contamination, including clinically important pathogens. In a randomized bench study, sterile chlorhexidine–alcohol and povidone-iodine immersion significantly reduced bioburden compared with ethanol or no intervention. When replacement is not feasible, chlorhexidine or iodine is preferable.

## Introduction

The so-called “five-second rule” has been debunked in food safety, yet a similar mindset persists in surgical environments.^1,2^ Implant drops are not uncommon with 61.5% occurring during emergency procedures.^3^ Despite the frequency, guidelines for managing dropped implants have not been established.

Studies of operating room (OR) contamination consistently demonstrate that surfaces, including floors, are frequently colonized by pathogens, making any dropped item, particularly an implant, potentially dangerous. Data from Weber et al, among others, underscore the real risk posed by contaminated OR environments.^4,5^

A 2019 literature review by Vautrin et al examined expert responses to dropped polyethylene (PE) implants and found that more than half of experienced orthopedic surgeons would prefer to delay definitive implantation rather than use a contaminated liner, underscoring that replacement is generally favored when feasible. However, other strategies included soaking the implant in antiseptic solutions or temporarily using a provisional implant.^6^

This study was designed to simulate a real-world scenario and assess the efficacy of 3 disinfectants on PE liners dropped on OR floors. The primary objective was to compare bacterial contamination counts before and after immersion in sterile preparations of chlorhexidine-alcohol (CHG), povidone-iodine (PI), or ethanol (EtOH).

## Methods

We conducted a prospective, randomized controlled bench study at Duke University Medical Center, a tertiary care hospital in Durham, North Carolina, between June and July 2025. The study was performed in 4 ORs primarily assigned to orthopedic surgery and occurred during orthopedic operations.

### OR cleaning practices

Floors were mopped between cases with Virex II 256 (Diversey Holdings, Fort Mill, SC) and again during overnight terminal cleaning. Tru-D SmartUVC disinfection was used after isolation cases and during terminal cleaning.

### Study procedures

Polyethylene (PE) liners from primary hip or knee arthroplasty cases were aseptically and gently placed on the OR floor immediately following a primary arthroplasty case, where the surgeon had stood during surgery, for 10 seconds. The surface of each liner was divided using a previously validated method into left and right halves: the left half was swabbed before intervention, the right half after intervention.^7^ Liners were randomized (1:1:1:1) to control (no disinfection), chlorhexidine-alcohol (2% CHG in 70% isopropyl alcohol), povidone-iodine (10%), or ethanol (70%); all disinfectants were sterile. Floor sponge samples were collected from drop sites to assess baseline bioburden after liners were dropped.

### Microbiological methods

All samples were processed and plated on standard microbiological media previously descibed.^7^ Colony-forming units (CFUs) were counted overall and at the species level, and organisms were identified by 16S rRNA sequencing. Clinically important pathogens (CIP) were defined as *Staphylococcus aureus*, *Enterococcus* species, and Gram-negative bacteria.

### Data analyses

The primary outcome was total CFU from liner surfaces after intervention. The secondary outcome was the proportion of liners contaminated with CIP after intervention. CFU counts were compared using Kruskal–Wallis tests with pairwise Mann–Whitney *U* tests for pairwise comparisons. Proportions were compared using *χ* ^2^ tests. Analyses were performed in SAS (version 9.4M9; SAS Institute, Cary, NC). A 2-tailed *P* < .05 was considered statistically significant. The study was determined exempt by the Duke University Health System IRB.

## Results

A total of 213 polyethylene liners were analyzed, including 142 hip and 71 knee liners, randomized to control (35 hip, 21 knee), ethanol (31 hip, 22 knee), chlorhexidine–alcohol (38 hip, 16 knee), or povidone-iodine (38 hip, 12 knee).

### OR floor sampling

Nineteen sponge samples were collected from OR floor drop sites. Median (IQR) total CFU was 2,958 (0–8,250), and all 19 samples yielded growth. Median MRSA/MSSA CFU was 0 (0–205), recovered from 8 (42%) samples. Median *Enterococcus* CFU was 3,690 (915–36,000), present in 18 (95%) samples. Median CFU Gram-negative spp. was 399 (0–4,450), recovered from 12 (63%) samples.

### Liner bioburden

Overall median (IQR) preintervention bioburden was 10 (0–60) CFU, decreasing to 0 (0–20) CFU postintervention (Table 1). CHG and PI performed similarly (*P* = .57) and both reduced CFU compared with control (*P* = .0003 and *P* = .0006, respectively). Ethanol did not differ from control (*P* = .12) and was less efficacious than CHG (*P* = .01) and PI (*P* = .03). In knee liners, all disinfectants reduced CFU versus control; in hip liners, CHG and PI were superior to control, ethanol was not (Table 1).

**Table 1.** Median bioburden of polyethylene liners before and after disinfection across study arms

	Overall		Control		Ethanol		CHG		Iodine		*P* values						
Timing Relative to Disinfection	Before	After	Before	After	Before	After	Before	After	Before	After	All Arms	CHG vs control	CHG vs PI	CHG vs ethanol	Control vs PI	Control vs ethanol	PI vs ethanol
Bioburden, Median (IQR)																	
Overall	10 (0–60)	0 (0–20)	15 (0–55)	15 (0–95)	10 (0–70)	10 (0–20)	0 (0–30)	0 (0–0)	10 (0–117)	0 (0–10)	<.001	<.001	.57	.01	<.001	.12	.03
Knee Liners	0 (0–50)	0 (0–20)	10 (0–160)	20 (0–260)	5 (0–47)	0 (0–20)	0 (0–32)	0 (0–0)	0 (0–20)	0 (0–10)	.01	.004	.32	.15	.04	.05	.58
Hip Liners	10 (0–77)	0 (0–20)	20 (0–40)	10 (0–70)	10 (0–95)	10 (0–25)	5 (0–30)	0 (0–10)	25 (10–175)	0 (0–10)	.008	.02	.99	.02	.01	.7	.01

*Note:* Data are presented as median (interquartile range) colony-forming units (CFU) for all polyethylene liners combined and stratified by knee and hip implants before and after disinfection. Study arms include control, ethanol, chlorhexidine-alcohol (CHG), and povidone-iodine (PI). *P* values for “All Arms” represent overall comparisons across all 4 study arms.

### Clinically important pathogens

Before intervention, CIPs were recovered from 73/213 liners (34%), decreasing to 41/213 (19%) postintervention (*P* = .004). CHG and PI reduced pathogen recovery compared with control (*P* = .003 and = .03, respectively). Ethanol did not differ from control or PI (*P* = .50 and *P* = .17, respectively) and was less efficacious than CHG (*P* = .03) (Table 2).

**Table 2.** Presence of clinically important pathogens (CIP) and specific organisms before and after disinfection across study arms

	Overall		Control		Ethanol		CHG		Iodine		*P* values						
Timing relative to disinfection	Before	After	Before	After	Before	After	Before	After	Before	After	All Arms	CHG vs control	CHG vs PI	CHG vs ethanol	Control vs PI	Control vs ethanol	PI vs ethanol
EIP																	
Overall	73 (34)	41 (19)	21 (38)	18 (32)	20 (38)	13 (25)	13 (24)	4 (7)	19 (38)	6 (12)	.004	.003	.64	.03	.03	.5	.17
Knee	25 (35)	18 (25)	7 (33)	7 (33)	9 (41)	7 (32)	6 (38)	3 (19)	3 (25)	1 (8)	.82	–	–	–	–	–	–
Hip	18 (34)	23 (16)	14 (40)	11 (31)	11 (35)	6 (19)	7 (18)	1 (3)	16 (42)	5 (13)	.009	.003	.2	.06	.11	.4	.71
S. aureus																	
Overall	47 (22)	25 (12)	14 (25)	10 (18)	12 (23)	10 (19)	7 (13)	1 (2)	14 (28)	4 (8)	.03	.007	.11	.01	.24	.91	.21
Knee	15 (21)	10 (14)	4 (19)	4 (19)	5 (23)	5 (23)	4 (25)	1 (6)	2 (17)	0 (0)	.36	–	–	–	–	–	–
Hip	32 (23)	15 (11)	10 (29)	6 (17)	7 (23)	5 (16)	3 (8)	0 (0)	12 (32)	4 (11)	.03	.03	.05	.03	.6	.93	.76
Enterococcus spp.																	
Overall	34 (16)	18 (8)	9 (16)	9 (16)	8 (15)	3 (6)	8 (15)	3 (6)	9 (18)	3 (6)	.13	–	–	–	–	–	–
Knee	13 (18)	9 (13)	5 (24)	4 (19)	4 (18)	2 (9)	3 (19)	2 (12)	1 (8)	1 (8)	.79	–	–	–	–	–	–
Hip	21 (15)	9 (6)	4 (11)	5 (14)	4 (13)	1 (3)	5 (13)	1 (3)	8 (21)	2 (5)	.28	–	–	–	–	–	–
Gram-negative spp.																	
Overall	25 (12)	6 (3)	10 (18)	6 (11)	5 (9)	0 (0)	5 (9)	0 (0)	5 (10)	0 (0)	.03	.03	1.0	1.0	.03	.03	1.0
Knee	7 (10)	3 (4)	4 (19)	3 (14)	2 (9)	0 (0)	1 (6)	0 (0)	0 (0)	0 (0)	.25	–	–	–	–	–	–
Hip	18 (13)	3 (2)	6 (17)	3 (9)	3 (10)	0 (0)	4 (11)	0 (0)	5 (13)	0 (0)	.24	–	–	–	–	–	–

*Note:* Data are presented as No. (%) of polyethylene liners positive for environmental implant pathogens (EIP), *Staphylococcus aureus*, *Enterococcus* species, or Gram-negative species before and after disinfection. Values are shown for all liners combined and stratified by knee and hip liners. Study arms include control, ethanol, chlorhexidine-alcohol (CHG), and povidone-iodine (PI). *P* values represent comparisons of the change in contamination between arms, calculated using *χ* ^2^ tests for overall comparisons and selected pairwise comparisons.

For *S. aureus*, overall prevalence declined from 22% to 12% (*P* = .03). After disinfection, CHG was more efficacious than control (*P* = .007) and ethanol (*P* = .01), with similar performance to iodine (*P* = .11). For *Enterococcus spp.*, overall prevalence dropped from 16% to 8%, but differences between arms were not statistically significant (overall *P* = .13). Gram-negative bacteria decreased from 12% to 3% overall (*P* = .03). All 3 interventions eliminated gram-negatives postdisinfection outperforming control (*P* = .03), but no difference was detected among them. By implant type, knees showed no significant between-arm differences for CIPs or individual pathogen groups, while hips mirrored the overall trends (Table 2).

## Discussion

In this randomized controlled study, both CHG and PI immersion reduced the bioburden on PE liners dropped on the OR floor compared to ethanol or control. These reductions extended to CIPs, with CHG and PI lowering postdisinfection recovery compared with control, whereas ethanol did not. Effects were clearest in hip liners as the absence of between-disinfectant differences in knees likely reflects smaller subgroup sample size rather than true equivalence. At the organism level, *S. aureus* declined with CHG and PI, *Enterococcus* decreased overall without clear between-arm differences, and Gram-negative organisms were eliminated postdisinfection by all 3 interventions, but not by control. Lastly, floor sponges demonstrated substantial baseline contamination underscoring the OR floor as a reservoir for implant contamination.

These findings align with prior work demonstrating the effectiveness of chlorhexidine-based disinfection. Mat-Salleh et al reported CHG superiority (5.4% positive cultures) compared with PI (67.6%) and ethanol (81.1%) for bone graft decontamination.^8^ In our study of polyethylene liners under operating room conditions, CHG and PI demonstrated comparable performance. This difference is likely due to (1) the use of a binary outcome (growth vs no growth), which is less granular than this study’s use of CFU counts and may obscure partial reductions in bioburden, (2) a lack of predisinfection samples so changes in CFU or specific pathogens were unable to be assessed, and (3) the study was underpowered to assess PI performance, with a borderline *P* value (=.059).

Importantly, only sterile disinfectant solutions should be used for implant decontamination as non-sterile agents have been associated with healthcare-associated infection outbreaks.^9,10^ Even with CHG or PI, disinfection did not guarantee sterility, indicating residual risk. Therefore, a dropped PE liner should be replaced. If an alternative is unavailable, immersion in sterile CHG or PI is preferable to ethanol or no intervention. Patients should be informed of the event and monitored for signs of infection. Other devices or tissues dropped in the OR may also become contaminated, with risk and cleaning effectiveness varying by material.

Future studies should explore prolonged disinfection times, alternative agents, or adjunctive technologies like ultraviolet disinfection chambers. Standardized protocols for intraoperative implant rescue may reduce uncertainty and improve patient safety.
